# Predictive models for chronic kidney disease after radical or partial nephrectomy in renal cell cancer using early postoperative serum creatinine levels

**DOI:** 10.1186/s12967-021-02976-2

**Published:** 2021-07-16

**Authors:** Dongwoo Chae, Na Young Kim, Ki Jun Kim, Kyemyung Park, Chaerim Oh, So Yeon Kim

**Affiliations:** 1 Department of Pharmacology, Yonsei University College of Medicine, 50-1 Yonsei-ro, Seodaemun-gu, Seoul, 03722 Republic of Korea; 2 Department of Anesthesiology and Pain Medicine, Anesthesia and Pain Research Institute, Yonsei University College of Medicine, 50-1 Yonsei-ro, Seodaemun-gu, Seoul, 03722 Republic of Korea

**Keywords:** Chronic kidney disease, Creatinine, Nephrectomy, Predictive factors, Renal cell cancer

## Abstract

**Background:**

Several predictive factors for chronic kidney disease (CKD) following radical nephrectomy (RN) or partial nephrectomy (PN) have been identified. However, early postoperative laboratory values were infrequently considered as potential predictors. Therefore, this study aimed to develop predictive models for CKD 1 year after RN or PN using early postoperative laboratory values, including serum creatinine (SCr) levels, in addition to preoperative and intraoperative factors. Moreover, the optimal SCr sampling time point for the best prediction of CKD was determined.

**Methods:**

Data were retrospectively collected from patients with renal cell cancer who underwent laparoscopic or robotic RN (n = 557) or PN (n = 999). Preoperative, intraoperative, and postoperative factors, including laboratory values, were incorporated during model development. We developed 8 final models using information collected at different time points (preoperative, postoperative day [POD] 0 to 5, and postoperative 1 month). Lastly, we combined all possible subsets of the developed models to generate 120 meta-models. Furthermore, we built a web application to facilitate the implementation of the model.

**Results:**

The magnitude of postoperative elevation of SCr and history of CKD were the most important predictors for CKD at 1 year, followed by RN (compared to PN) and older age. Among the final models, the model using features of POD 4 showed the best performance for correctly predicting the stages of CKD at 1 year compared to other models (accuracy: 79% of POD 4 model versus 75% of POD 0 model, 76% of POD 1 model, 77% of POD 2 model, 78% of POD 3 model, 76% of POD 5 model, and 73% in postoperative 1 month model). Therefore, POD 4 may be the optimal sampling time point for postoperative SCr. A web application is hosted at https://dongy.shinyapps.io/aki_ckd.

**Conclusions:**

Our predictive model, which incorporated postoperative laboratory values, especially SCr levels, in addition to preoperative and intraoperative factors, effectively predicted the occurrence of CKD 1 year after RN or PN and may be helpful for comprehensive management planning.

**Supplementary Information:**

The online version contains supplementary material available at 10.1186/s12967-021-02976-2.

## Background

Acute kidney injury (AKI) is a common complication after nephrectomy in renal cell cancer (RCC), with radical nephrectomy (RN) associated with a noticeably higher risk than partial nephrectomy (PN) [1, 2]. AKI is defined as an abrupt decrease in kidney function occurring over 7 days or less, whereas chronic kidney disease (CKD) is defined by the persistence of kidney disease for a period of > 90 days [3]. The severity of AKI and recovery time have been implicated as important predictors of CKD progression [3–6]. Surgically induced CKD may be associated with a lower risk of progression and mortality than CKD due to medical causes [7]. However, despite this, an estimated glomerular filtration rate (eGFR) less than 45 mL/min/1.73 m^2^ in patients with surgically induced CKD has been associated with an increased risk of mortality [8].

Numerous studies have investigated the predictive factors for CKD following RN or PN [9–16], some of which developed predictive models for CKD [13, 14]. However, these studies analyzed preoperative and intraoperative factors (such as patient characteristics, preoperative laboratory values, and surgical type or technique) as possible predictors, without including postoperative laboratory values [9–14]. Although few studies included the occurrence of AKI or time to nadir eGFR as one of the predictors of CKD [15–17], serial changes of serum creatinine (SCr) was not considered. When considering the variable trajectories following AKI [18], postoperative laboratory values, especially SCr, should be considered for better prediction. Therefore, we hypothesized that SCr levels collected in the first 5 days after nephrectomy would provide important information to predict the SCr levels 1 year after surgery and ultimately, the occurrence of CKD. Thus, this study aimed to develop predictive models for CKD after RN or PN using early postoperative laboratory values, including SCr levels, in addition to preoperative and intraoperative factors, and build a web application to facilitate their implementation. Moreover, we aimed to find optimal SCr sampling time points for accurate CKD prediction.

## Methods

### Patients

The analysis data set included 1,556 patients with RCC who received either laparoscopic or robotic RN (n = 557) or PN (n = 999) between December 2005 and May 2019 and were at least followed up to 1 year after surgery. Patients lost to follow up or died before 1 year were excluded. Data were retrospectively collected from the electronic medical records of a single institution.

### Features used for prediction

Our study aimed to predict the rise in SCr levels relative to preoperative value at 1 year after surgery (between 11 and 13 months after surgery), hereafter denoted as $\Delta {SCr}_{1y}$, given the following features:

(i) Patient characteristics: age, sex, weight, history of diabetes mellitus, history of hypertension, history of CKD, and tumor size.
(ii) Preoperative and intraoperative factors: preoperative complete blood count (CBC; hematocrit, neutrophil, lymphocyte, monocyte, and platelet counts), preoperative routine chemistry (SCr, blood urea nitrogen [BUN], uric acid, total protein, albumin, aspartate aminotransferase, alanine aminotransferase [ALT], alkaline phosphatase, total bilirubin, and cholesterol), preoperative serum electrolytes (sodium, potassium, calcium, phosphate), preoperative vital signs (systolic and diastolic blood pressure [BP], and heart rate), size of mass removed, duration of anesthesia, and bleeding amount.
(iii) Postoperative factors: baseline (preoperative) subtracted SCr, CBC, routine chemistry, serum electrolytes, and vital signs immediately after surgery, postoperative day (POD) 1–5, and postoperative 1 month

Data were also collected on POD 7 and 14 and at 3 and 6 months; however, they were not included in the model development. For brevity, a common notation involving $\Delta$ has been used throughout to represent the baseline-subtracted level of different variables, with the associated subscript indicating the sampling time point. For example, baseline subtracted SCr on POD 3 has been denoted as $\Delta {SCr}_{3d}$. The letters *d*, *m*, and *y*, used as subscripts, represent the day, month, and year, respectively.

Missing values were imputed using multiple imputation by chained equations (MICE), also known as fully conditional specification or sequential regression multiple imputation. The method operates under the assumption that missing data are Missing at Random (MAR), i.e., the probability of a particular value being missing depends only on the observed values and not the unobserved values [19]. Since missing values in our data showed a clear time-dependency and were likely unrelated to the true value of SCr, we assumed that the condition of MAR was fulfilled, thus allowing the partial deduction of the missing values based on the measurements immediately before and after them. The highest proportions of missing values occurred on POD 4 and 5 in both RN and PN (Additional file 1: Fig. S1). The proportions of patients without any missing value were 14.7% and 16.8% in RN and PN, respectively. The widely validated R package, *mice,* was used to carry out the imputation process [20].

### Model development

First, the features were grouped into eight categories, namely ${F}_{pre}$, ${F}_{0d}$, ${F}_{1d}$, ${F}_{2d}$, ${F}_{3d}$, ${F}_{4d}$, ${F}_{5d}$, and ${F}_{1m}$, based on their time of acquisition. ${F}_{pre}$ only included factors available prior to completion of the surgery, mentioned above as patient characteristics and preoperative and intraoperative factors. Other feature sets, denoted as ${F}_{i}$ with i = 0, 1, 2, 3, 4, 5 days, included ${F}_{pre}$ and information collected on the ${i}^{th}$ postoperative day. The last feature set, ${F}_{1m}$, included ${F}_{pre}$ and features collected 1 month after surgery. Thereafter, Lasso regression models were built on each of the feature sets to predict $\Delta {SCr}_{1y}$, hereafter referred to individually as ${Model}_{Lasso,pre}$, ${Model}_{Lasso,0d}$, ${Model}_{Lasso,1d}$, ${Model}_{Lasso,2d}$, ${Model}_{Lasso,3d}$, ${Model}_{Lasso,4d}$, ${Model}_{Lasso,5d}$, and ${Model}_{Lasso,1m}$, and collectively, as ${Model}_{Lasso}$. The features with non-zero regression coefficients in ${Model}_{Lasso}$ were collectively referred to as ${F}_{Lasso}$ and individually as ${F}_{Lasso,pre}$, ${F}_{Lasso,0d}$, ${F}_{Lasso,1d}$, ${F}_{Lasso,2d}$, ${F}_{Lasso,3d}$, ${F}_{Lasso,4d}$, ${F}_{Lasso,5d}$, and ${F}_{Lasso,1m}$; each of these consisted of selected features from ${F}_{pre}$, ${F}_{0d}$, ${F}_{1d}$, ${F}_{2d}$, ${F}_{3d}$, ${F}_{4d}$, ${F}_{5d}$, and ${F}_{1m}$, respectively.

Prior to model development, we split the dataset into training and test datasets in the ratio of 8:2. The features were z-score normalized. A grid search algorithm and four-fold cross-validation were used on the training dataset to tune the shrinkage hyper-parameters.

### Construction of the final model

To retain only the most parsimonious set of features, we calculated the Spearman’s partial correlation coefficients [21] of ${F}_{Lasso}$ with $\Delta {SCr}_{1y}$ and eliminated features with absolute values less than 0.1. This yielded the final selected features, collectively referred to as ${F}_{final}$ and individually as ${F}_{final,pre}$, ${F}_{final,0d}$, ${F}_{final,1d}$, ${F}_{final,2d}$, ${F}_{final,3d}$, ${F}_{final,4d}$, ${F}_{final,5d}$, and ${F}_{final,1m}$. We then constructed multivariate linear regression models using ${F}_{final}$. Unlike the Lasso models, the features were not z-score normalized, so the estimated regression coefficients could be readily interpreted. The final models were referred to as ${Model}_{final,pre}$, ${Model}_{final,0d}$, ${Model}_{final,1d}$, ${Model}_{final,2d}$, ${Model}_{final,3d}$, ${Model}_{final,4d}$, ${Model}_{final,5d}$, and ${Model}_{final,1m}$.

**Fig. 1:**
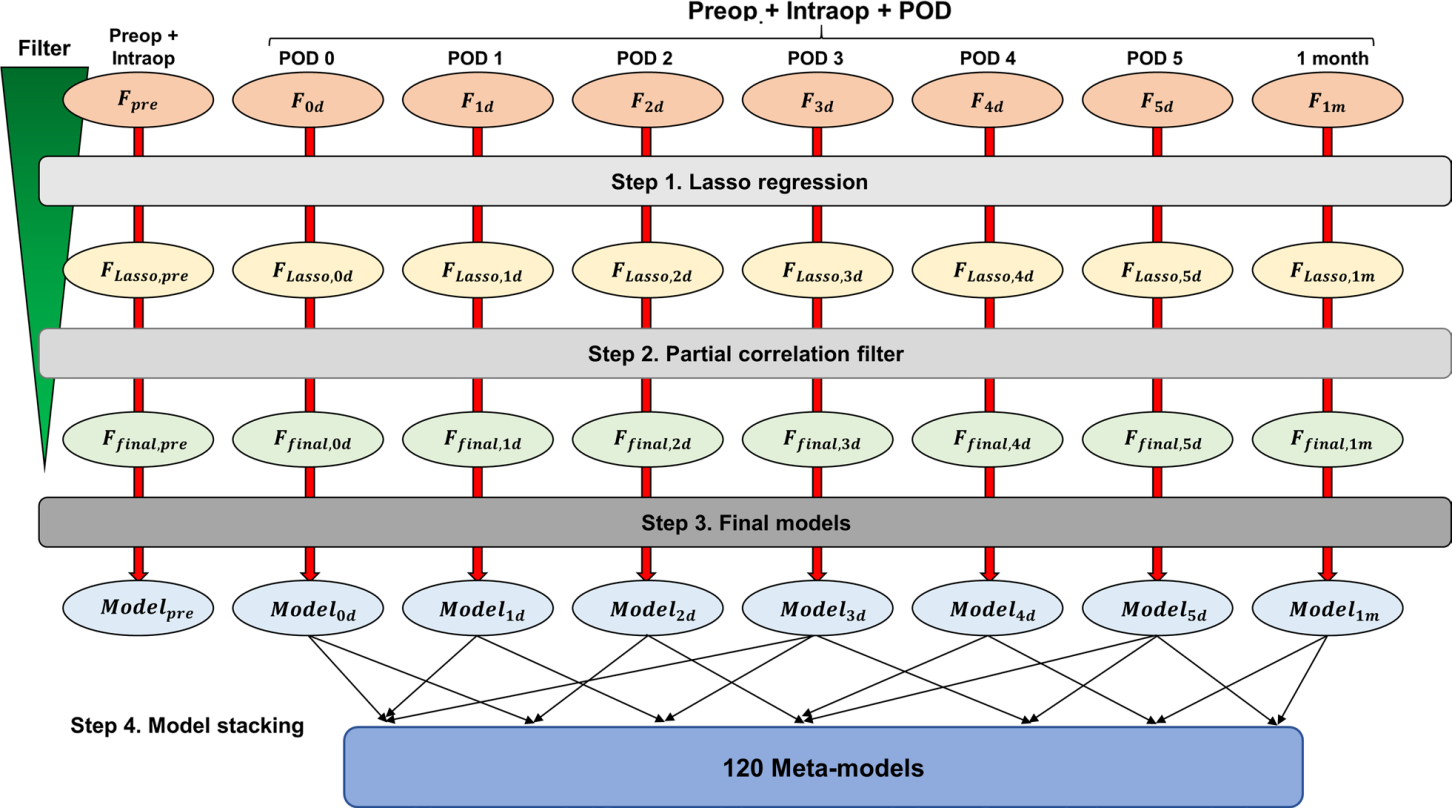
Schematic diagram of the overall analysis workflow. Candidate features were first grouped into preoperative, intraoperative, and postoperative feature sets. Preoperative and intraoperative features were merged into a set of ${F}_{pre}$, which were used in all the tested models. Postoperative features were categorized based on the sampling time points (postoperative day [POD] 0, 1, 2, 3, 4, 5, and postoperative 1 month). Each of these feature sets was combined with ${F}_{pre}$ to yield 7 different feature sets (${F}_{0d}$, …, ${F}_{5d}$, and ${F}_{1m}$). The 8 feature sets were used to fit 8 different Lasso regression models. Features of each set with non-zero coefficients, ${F}_{Lasso}$, were then passed onto a partial correlation filter that evaluated the correlation of each of the features with the target variable $\Delta {SCr}_{1y}$. The final features, ${F}_{final}$, were then used to train the final multivariate linear regression models. The final step used all possible combinations of the predictions generated by the 7 final models, ${Model}_{0d-1m}$, to yield 120 meta-models

The predictive performances of ${Model}_{final}$ were then compared with those of ${Model}_{Lasso}$. ${R}^{2}$ and mean squared error (MSE) between the predicted and observed $\Delta {SCr}_{1y}$ values calculated using the test dataset were used as the performance metrics. The eGFR was calculated using the Chronic Kidney Disease Epidemiology Collaboration (CKD-EPI) equation [22]. CKD was categorized according to the eGFR: stage 1 (≥ 90 mL/min/1.73 m^2^), stage 2 (60–89 mL/min/1.73 m^2^), and stage 3 and higher (< 60 mL/min/1.73 m^2^) [23]. The overall analysis workflow is schematically shown in Fig. 1.

### Model stacking

We performed model stacking by developing meta-models using predictions generated from all possible subsets of ${Model}_{final,0d}$, ${Model}_{final,1d}$, ${Model}_{final,2d}$, ${Model}_{final,3d}$, ${Model}_{final,4d}$, ${Model}_{final,5d}$, and ${Model}_{final,1m}$ as features. Ridge regressions with four-fold cross-validation were used to acquire appropriate weights to be assigned to each of the predictions generated by the component models. A total of 120 (= ${2}^{7}$– 1–7) meta-models were thus developed. Meta-models trained using predictions of k different final models (k = 2, …, 7) are hereafter be referred to as ${Meta\_models}_{k}$. For example, a meta-model developed using predictions of ${Model}_{final,0d}$, ${Model}_{final,3d}$, and ${Model}_{final,4d}$ constituted one of ${Meta\_models}_{3}$, and was used when supplied with SCr and laboratory values measured on POD 0, 3, and 4.

### Web application development

To facilitate the automatic selection and implementation of the meta-model, we developed a web application with a user-friendly interface, hosted at https://dongy.shinyapps.io/aki_ckd. The *shiny* package in R (https://shiny.rstudio.com) was used for programming the application. The application used 8 basic models (${Model}_{final,pre}$, ${Model}_{final,0d}$, ${Model}_{final,1d}$, ${Model}_{final,2d}$, ${Model}_{final,3d}$, ${Model}_{final,4d}$, ${Model}_{final,5d}$, and ${Model}_{final,1m}$) and 120 meta-models built from their predictions. Given only preoperative and intraoperative factors, ${Model}_{final,pre}$ is activated to generate the predictions. Following the input of extra postoperative factors, the optimal model is chosen based on the number of samples (= k) and the corresponding PODs of their acquisition. Outputs of the model are predicted values of $\Delta {SCr}_{1y}$, eGFR, and CKD stage at 1 year.

## Results

Table 1 summarizes the baseline characteristics of patients that underwent RN and PN. The longitudinal trajectories of postoperative SCr in RN and PN are shown in Additional file 2: Fig. S2. SCr typically increased from POD 0 to POD 3, decreased from POD 4 to 7, showed a secondary surge until POD 15, and gradually declined towards the final level. SCr levels on POD 4 and 5 showed the highest correlation with SCr level at 1 year (Additional file 3: Table S1). In RN and PN, 43.1% (240 of 557) and 7.2% (72 of 999) of the patients, respectively, developed CKD stage 3 and higher 1 year after surgery. All the above exploratory analyses were carried out using the raw data prior to imputation.

**Table 1:** Patient characteristics

Variable	Radical nephrectomy (n = 557)	Partial nephrectomy (n = 999)
Age (years)	56 (12)	54 (12)
Male sex	364 (67%)	668 (67%)
ASA physical status		
I	116 (21%)	299 (30%)
II	318 (57%)	588 (59%)
III	123 (22%)	112 (11%)
Medical history		
Hypertension	256 (46%)	381 (38%)
Diabetes mellitus	98 (18%)	156 (16%)
Chronic renal disease	6 (< 1%)	8 (< 1%)
Tumor size (cm)	5.2 (2.3)	2.6 (1.4)
Size of mass removed (cm)	13.4 (3.3)	4.4 (2.2)
R.E.N.A.L. nephrectomy score	–	6.0 (1.8)
Warm ischemia time (min)	–	23.4 (12.6)
Duration of anesthesia (min)	207 (58)	224 (53)
Bleeding amount (mL)	171 (380)	232 (279)
Preoperative laboratory values		
Hematocrit (%)	41.4 (4.6)	42.5 (4.3)
Neutrophil count (10^3^/μL)	4.31 (1.74)	3.77 (1.51)
Lymphocyte count (10^3^/μL)	1.99 (0.63)	1.99 (0.61)
Monocyte count (10^3^/μL)	0.44 (0.17)	0.42 (0.15)
Platelet count (10^3^/μL)	264 (85)	252 (67)
Creatinine (mg/dL)^a^	0.88 (0.25)	0.85 (0.21)
Blood urea nitrogen (mg/dL)^a^	14.4 (4.4)	14.2 (3.9)
Uric acid (mg/dL)^a^	5.1 (1.4)	5.2 (1.4)
Total protein (g/dL)^a^	7.3 (0.5)	7.3 (0.5)
Albumin (g/dL)^a^	4.5 (0.4)	4.6 (0.4)
Aspartate aminotransferase (IU/L)^a^	22 (11)	23 (11)
Alanine aminotransferase (IU/L)^a^	23 (16)	25 (16)
Alkaline phosphatase (IU/L)^a^	68 (26)	65 (19)
Total bilirubin (mg/dL)^a^	0.7 (0.3)	0.7 (0.3)
Cholesterol (mg/dL)^a^	179 (42)	186 (36)
Sodium (mEq/L)^a^	141 (2)	141 (2)
Potassium (mEq/L)^a^	4.4 (0.4)	4.4 (0.4)
Calcium (mEq/L)^a^	9.4 (0.5)	9.4 (0.4)
Phosphate (mEq/L)^a^	3.6 (0.5)	3.5 (0.5)
Preoperative vital signs		
Systolic blood pressure (mmHg)	132 (14)	132 (16)
Diastolic blood pressure (mmHg)	81 (10)	83 (11)
Heart rate (beats/min)	74 (11)	74 (11)

Values represent the mean (SD) or number of patients (proportion). *ASA* American Society of Anesthesiologists, P*N* partial nephrectomy, *R.E.N.A.L.* radius, exophytic or endophytic, nearness to collecting system or sinus, anterior or posterior location, and location relative to polar lines

^a^Data were obtained from serum

### Model development

Lasso regression models—${Model}_{Lasso,pre}$, ${Model}_{Lasso,0d}$, ${Model}_{Lasso,1d}$, ${Model}_{Lasso,2d}$, ${Model}_{Lasso,3d}$, ${Model}_{Lasso,4d}$, ${Model}_{Lasso,5d}$, and ${Model}_{Lasso,1m}$, were developed using 8 different feature sets—${F}_{pre}$, ${F}_{0d}$, ${F}_{1d}$, ${F}_{2d}$, ${F}_{3d}$, ${F}_{4d}$, ${F}_{5d}$, and ${F}_{1m}$, respectively. We then identified subsets of the original feature sets that were associated with non-zero regression coefficients (i.e., ${F}_{Lasso}$). The features included in each subset of ${F}_{Lasso}$ and their estimated regression coefficients are shown in Additional file 3: Table S2.

The ${R}^{2}$ statistics of ${Model}_{Lasso,pre}$, ${Model}_{Lasso,0d}$, ${Model}_{Lasso,1d}$, ${Model}_{Lasso,2d}$, ${Model}_{Lasso,3d}$, ${Model}_{Lasso,4d}$, ${Model}_{Lasso,5d}$, and ${Model}_{Lasso,1m}$ were 0.432, 0.481, 0.507, 0.538, 0.562, 0.603, 0.589, and 0.630; their MSEs were 0.025, 0.023, 0.022, 0.021, 0.02, 0.018, 0.018, and 0.017, respectively. The best performing model was, as expected, the model using factors collected at 1 month (${Model}_{Lasso,1m}$). Among the models utilizing single time point information between POD 0 and 5, ${Model}_{Lasso,4d}$ showed the best predictive performance for $\Delta {SCr}_{1y}$. Overall, the models utilizing postoperative factors showed superior predictive performance than those using only preoperative and intraoperative factors (i.e., ${Model}_{Lasso,pre}$).

For all features included in each ${F}_{Lasso}$ subset, partial Spearman’s correlation coefficients with $\Delta {SCr}_{1y}$ were calculated; only features whose absolute values of the coefficients were greater than 0.1 (i.e., ${F}_{final}$) were retained. Final regression models (i.e., ${Model}_{final}$) were developed on ${F}_{final}$ and their estimation results are shown in Table 2.

**Table 2:** Regression coefficients of the final models sorted in the order of highest statistical significance

Model	Predictive performance	Feature	Estimates	t values	p values
${Model}_{final,pre}$	${R}^{2}$=0.444 MSE = 0.025	History of CKD	0.67	13.051	< 0.001
		Radical nephrectomy	0.188	8.684	< 0.001
		Male	0.06	5.243	< 0.001
		Size of mass removed (cm)	0.01	4.827	< 0.001
		Age (years)	0.0021	4.808	< 0.001
${Model}_{final,0d}$	${R}^{2}$=0.530 MSE = 0.021	History of CKD	0.722	14.938	< 0.001
		$\Delta {SCr}_{0d}$(mg/dL)	0.540	13.716	< 0.001
		Radical nephrectomy	0.2	9.754	< 0.001
		Age (years)	0.0033	7.771	< 0.001
		Size of mass removed (cm)	0.0055	2.769	0.006
${Model}_{final,1d}$	${R}^{2}$=0.505 MSE = 0.022	$\Delta {SCr}_{1d}$(mg/dL)	0.391	15.443	< 0.001
		History of CKD	0.632	13.193	< 0.001
		Radical nephrectomy	0.168	8.343	< 0.001
		Size of mass removed (cm)	0.0029	1.456	0.146
${Model}_{final,2d}$	${R}^{2}$=0.550 MSE = 0.02	$\Delta {SCr}_{2d}$(mg/dL)	0.414	20.76	< 0.001
		Radical nephrectomy	0.141	11.832	< 0.001
		History of CKD	0.471	10.157	< 0.001
		Age (years)	0.0022	5.537	< 0.001
${Model}_{final,3d}$	${R}^{2}$=0.575 MSE = 0.019	$\Delta {SCr}_{3d}$(mg/dL)	0.482	22.056	< 0.001
		History of CKD	0.44	9.716	< 0.001
		Radical nephrectomy	0.120	6.421	< 0.001
		Age (years)	0.0022	5.91	< 0.001
		Size of mass removed (cm)	0.0007	0.361	0.718
${Model}_{final,4d}$	${R}^{2}$=0.608 MSE = 0.018	$\Delta {SCr}_{4d}$(mg/dL)	0.449	17.84	< 0.001
		Radical nephrectomy	0.117	10.031	< 0.001
		History of CKD	0.441	9.735	< 0.001
		$\Delta {BUN}_{4d}$(mg/dL)	0.0083	7.062	< 0.001
		Preoperative BUN (mg/dL)	0.0070	4.913	< 0.001
		Age (years)	0.0015	3.732	< 0.001
${Model}_{final,5d}$	${R}^{2}$=0.565 MSE = 0.019	$\Delta {SCr}_{5d}$(mg/dL)	0.472	18.7	< 0.001
		History of CKD	0.573	12.658	< 0.001
		Radical nephrectomy	0.122	9.918	< 0.001
		$\Delta {BUN}_{5d}$(mg/dL)	0.0059	5.954	< 0.001
${Model}_{final,1m}$	${R}^{2}$=0.643 MSE = 0.016	$\Delta {SCr}_{1m}$(mg/dL)	0.638	26.397	< 0.001
		History of CKD	0.526	12.324	< 0.001
		Radical nephrectomy	0.079	6.565	< 0.001

*CKD* chronic kidney disease, *SCr* serum creatinine, *BUN* blood urea nitrogen, *d* day, *m* month, *MSE* mean squared error

**Fig. 2:**
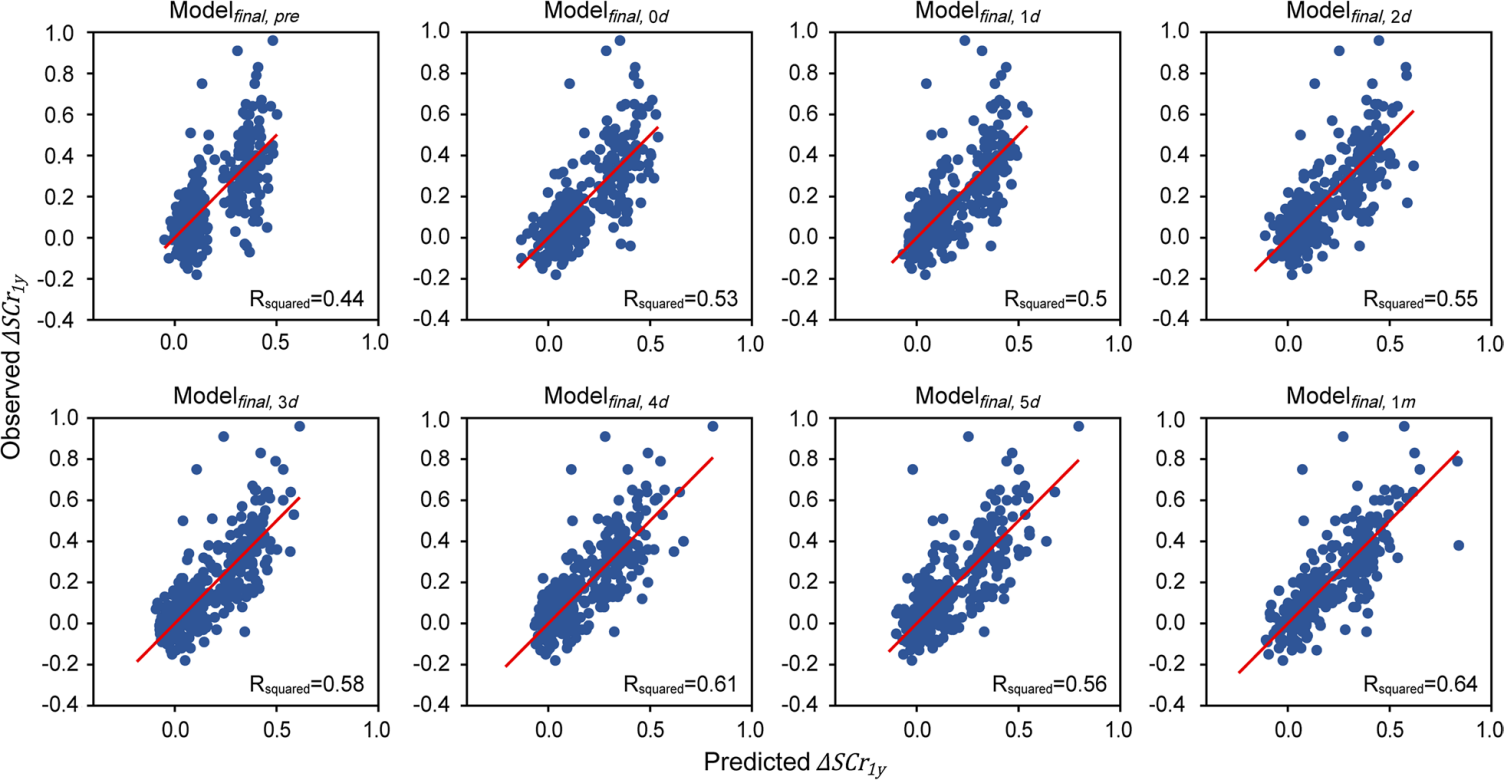
Goodness-of-fit plots of the 8 final models. The ordinate and abscissa represent the observed and predicted $\Delta {SCr}_{1y}$, respectively. The red lines indicate the lines of unity

The ${R}^{2}$ statistics of ${Model}_{final,pre}$, ${Model}_{final,0d}$, ${Model}_{final,1d}$, ${Model}_{final,2d}$, ${Model}_{final,3d}$, ${Model}_{final,4d}$, ${Model}_{final,5d}$, and ${Model}_{final,1m}$ were 0.444, 0.530, 0.505, 0.550, 0.575, 0.608, 0.565, and 0.643 and their MSEs were 0.025, 0.021, 0.022, 0.020, 0.019, 0.018, 0.019, and 0.016, respectively. Comparison of the predictive performances of ${Model}_{final}$ with ${Model}_{Lasso}$ suggested that ${Model}_{final}$ generally performed better than ${Model}_{Lasso}$, despite a fewer number of predictive features being included. Similar to ${Model}_{Lasso}$, the best prediction was achieved using ${F}_{final,1m}$, followed by ${F}_{final,4d,}$ The goodness-of-fit plots of observed vs. predicted values are shown in Fig. 2. The classification performances in terms of predicting the CKD stage are shown in Table 3. ${Model}_{final,4d}$ was found to confer the best accuracy, weighted averaged precision, and weighted averaged recall.

**Table 3:** Classification performances of chronic kidney disease stage based on the final models

		Predicted		
		Normal or Stage 1	Stage 2	Stage 3
$${Model}_{final,pre}$$				
Observed	Normal or Stage 1	77	32	0
	Stage 2	16	101	19
	Stage 3	0	17	49
Accuracy: 73%				
Weighted average precision: 74%				
Weighted average recall: 73%				
$${Model}_{final,0d}$$				
Observed	Normal or Stage 1	74	35	0
	Stage 2	12	108	16
	Stage 3	0	14	52
Accuracy: 75%				
Weighted average precision: 76%				
Weighted average recall: 75%				
$${Model}_{final,1d}$$				
Observed	Normal or Stage 1	81	28	0
	Stage 2	15	108	13
	Stage 3	0	18	48
Accuracy: 76%				
Weighted average precision: 77%				
Weighted average recall: 76%				
$${Model}_{final,2d}$$				
Observed	Normal or Stage 1	84	25	0
	Stage 2	14	105	17
	Stage 3	1	16	49
Accuracy: 77%				
Weighted average precision: 77%				
Weighted average recall: 77%				
$${Model}_{final,3d}$$				
Observed	Normal or Stage 1	83	26	0
	Stage 2	13	109	14
	Stage 3	1	13	52
Accuracy: 78%				
Weighted average precision: 79%				
Weighted average recall: 78%				
$${Model}_{final,4d}$$				
Observed	Normal or Stage 1	84	25	0
	Stage 2	16	110	10
	Stage 3	1	12	53
Accuracy: 79%				
Weighted average precision: 80%				
Weighted average recall: 79%				
$${Model}_{final,5d}$$				
Observed	Normal or Stage 1	81	28	0
	Stage 2	18	105	13
	Stage 3	1	16	49
Accuracy: 76%				
Weighted average precision: 76%				
Weighted average recall: 76%				
$${Model}_{final,1m}$$				
Observed	Normal or Stage 1	84	24	0
	Stage 2	14	107	15
	Stage 3	1	14	51
Accuracy: 73%				
Weighted average precision: 74%				
Weighted average recall: 73%				

Normal or stage 1, estimated glomerular filtration rate (eGFR) ≥ 90 mL/min/1.73 m^2^; stage 2, eGFR 60–89 mL/min/1.73 m^2^; stage 3, eGFR < 60 mL/min/1.73 m^2^. *d* day, *m* month

### Model stacking

Ridge regressions with zero intercept were performed using predictions of all possible subsets of ${Model}_{final}$, yielding 120 meta-models. The average performances of ${Meta\_model(s)}_{k}$ improved with increasing k. For k = 2, 3, 4, 5, 6, and 7, the mean ${R}^{2}$ statistics were 0.606, 0.624, 0.637, 0.649, 0.659, and 0.669, respectively. ${Meta\_model}_{7}$, which utilizes all 7 models (i.e.${Model}_{final,0d}$, ${Model}_{final,1d}$, ${Model}_{final,2d}$, ${Model}_{final,3d}$, ${Model}_{final,4d}$, ${Model}_{final,5d}$, and ${Model}_{final,1m}$) offered the best predictive performance. In particular, it outperformed ${Model}_{final,1m}$ (${R}^{2}$=0.630). ${Meta\_model}_{7}$ showed precisions of 90%, 74%, and 79% and recalls of 78%, 83%, and 76% for classifying CKD stages 1, 2, and 3, respectively (average accuracy, 80%; weighted average precision, 81%; weighted average recall, 80%).

### Web application development

**Fig. 3:**
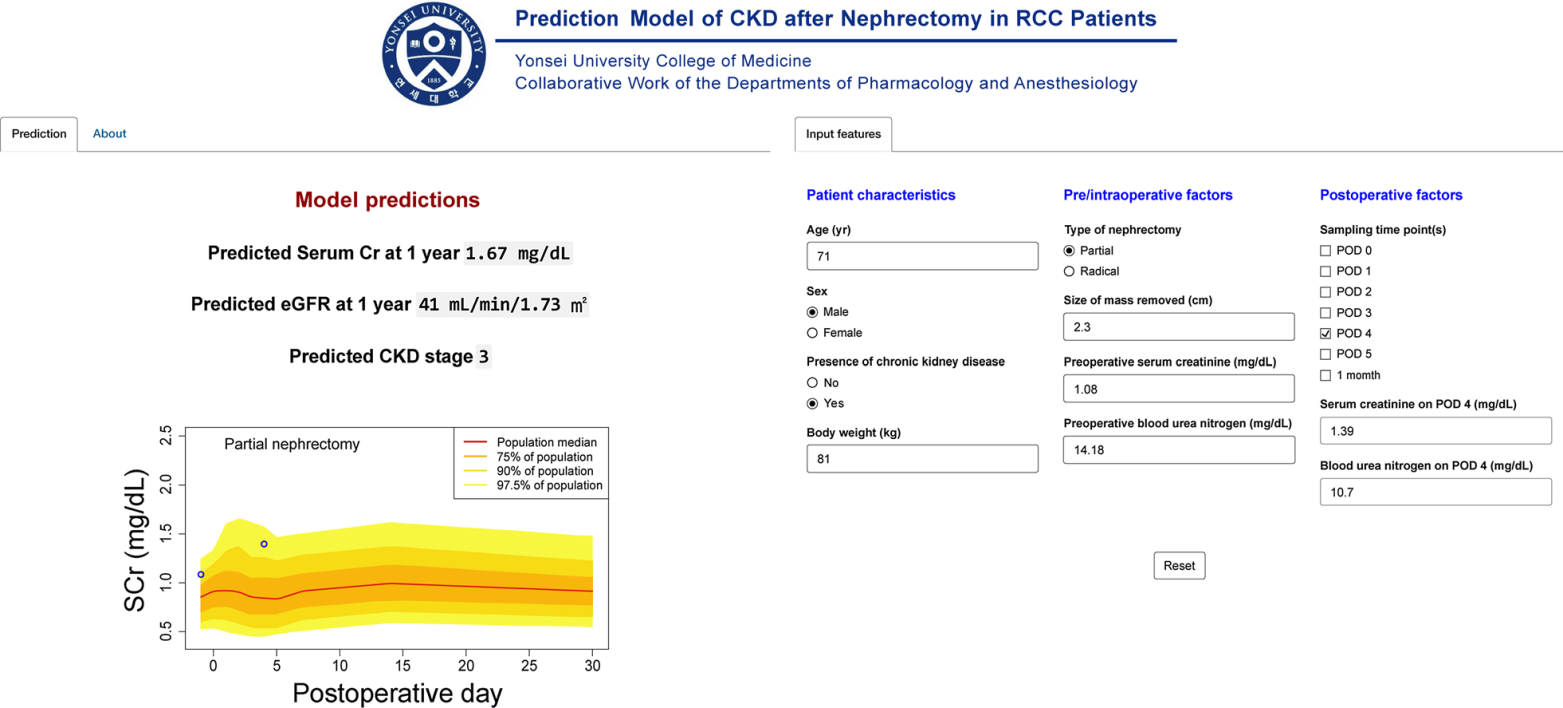
Screenshot of the developed web application (https://dongy.shinyapps.io/aki_ckd). The minimum required information were patient characteristics and preoperative and intraoperative factors. As postoperative measurements of SCr (and BUN if measured on postoperative days 4 and 5) become available, they can be entered into the newly appearing widget after clicking on the checkboxes under the sampling time point(s) heading. The predictions are updated in real-time with the incremental addition of newly acquired information. In the graph of SCr versus time, the different percentiles of longitudinal trajectories of SCr in our patients are shown as different shades of yellow-colored bands, median value as a red line, and SCr measurements as blue open circles

Figure 3 shows the screenshot of the developed web application. The minimum information required to run the application are patient characteristics, such as age, sex, history of CKD, and preoperative and intraoperative factors, including the type of nephrectomy (RN or PN), size of mass removed, and preoperative SCr and BUN levels. The left panel is used for generating predictions. As postoperative measurements of SCr become available, they can be used to update the predictions.

## Discussion

We developed predictive models for CKD 1 year after RN or PN that fully incorporated preoperative, intraoperative, and postoperative factors. Our work can be summarized as follows: 1) We clearly demonstrated the need to incorporate early postoperative information (specifically SCr levels) to accurately predict long-term renal function. 2) Within the first postoperative week we identified POD 4 as the optimal sampling point for SCr (and BUN). 3) We identified the magnitude of early SCr elevation, history of CKD, surgery type (RN or PN), and patient age as the most robust predictors of CKD. 4) We provide a practical framework to predict CKD and offer an easy-to-use web application to implement our models.

Although surgically induced CKD may have a better prognosis than CKD due to medical causes [7], the risk of mortality is known to increase if patients have a reduced eGFR (< 45 mL/min/1.73 m^2^) following RCC surgery [8]. Our results showed that 14% (78 out of 557) of RN patients and 2.2% (22 out of 999) of PN patients had an eGFR < 45 mL/min/1.73 m^2^ 1 year after surgery. This percentage was enough to warrant attention. Studies investigating the risk factors for CKD after RN or PN have shown older age, male sex, history of CKD, diabetes mellitus, and RN as independent predictors [9–15]. However, most of these studies did not consider perioperative laboratory values. Another study showed that time to nadir eGFR was one of the predictors of CKD [18]. However, computing the exact time to nadir eGFR requires intensive sampling, and may limit easy clinical implementation. Moreover, the perioperative laboratory values tested was limited only to eGFR in that study. In contrast to the aforementioned studies, our study comprehensively considered perioperative laboratory values, including CBC, routine chemistry, and serum electrolytes as predictive factors. In all the final models, an increase in SCr levels from the preoperative value and history of CKD were the most important features (Table 2). Among other features, RN (compared to PN) was robustly associated with a higher $$\Delta {SCr}_{1y}$$. Older age was additionally depicted as a significant risk factor in most final models ($${Model}_{final,pre}$$, $${Model}_{final,0d}$$, $${Model}_{final,2d}$$, $${Model}_{final,3d}$$, and $${Model}_{final,4d}$$). Male sex was only significant in $${Model}_{final,pre}$$, which was built using just preoperative information; its effect was fully accounted for by other factors once postoperative information was available. The size of mass removed was a significant feature only immediately after surgery ($${Model}_{final,0d})$$, and its contribution disappeared after incorporating the SCr values on POD 1 and onwards. Overall, the important predictors for CKD occurrence were postoperative SCr levels, history of CKD, RN, and older age. Most other factors such as postoperative electrolytes, CBC, routine chemistry, and vital signs that were tested as candidate predictors were insignificant.

In addition to model development, we aimed to identify the optimal time point of postoperative SCr sampling for predicting CKD. To this end, we compared the predictive performances of 6 final models that used the information obtained on POD 0 to 5 (${Model}_{final,0d}$−${Model}_{final,5d}$) to that of a reference model that only used preoperative information (${Model}_{final,pre}$), and then to that of a model that used information collected at 1 month (${Model}_{final,1m}$). The predictive performances of ${Model}_{final,0d}-{Model}_{final,5d}$ were better than those of ${Model}_{final,pre}$ but were almost similar to those of ${Model}_{final,1m}$. Hence, postoperative SCr levels measured in the first 5 days after surgery constituted crucial, nearly sufficient information to predict CKD at 1 year. Among ${Model}_{final,0d}$−${Model}_{final,5d}$, ${Model}_{final,4d}$ showed the best performance. In predicting the CKD stage, this model demonstrated classification accuracy of 79%, weighted averaged precision of 80%, and weighted averaged recall of 79% (Table 3). This suggested that POD 4 may be the optimal sampling point for predicting CKD.

To maximize the predictive performance, we adopted model stacking, a technique increasingly used in the medical field [24–26], wherein predictions from each of the 7 models (excluding the ${Model}_{final,pre}$) were combined in all possible ways to generate 120 feature sets. Ridge regression models were then trained on these sets, yielding 120 meta-models. Our prediction strategy was to select from the meta-models, the one that makes best use of all available information. For example, if we had SCr measurements on POD 3, 4, and 5, we would choose a meta-model built on predictions of ${Model}_{final,3d}$, ${Model}_{final,4d}$, and ${Model}_{final,5d}$.

This study has a few limitations. First, the data used for model building were retrospectively collected at a single center primarily comprising Korean patients. Hence, for generalization to patients of different ethnic backgrounds or those treated under different hospital environments, external validation is required. Second, this study only included surgeries that used minimally invasive laparoscopic or robotic techniques, and not open techniques. One study reported a lower risk of CKD in minimally invasive approaches [14], whereas others reported a similar risk [10, 13]. However, minimally invasive approaches are being used more frequently for RN and PN [27]; thus, our model may be appropriate for future studies. Third, SCr was used as the surrogate of postoperative renal function and the target to be predicted, although the definition of CKD is based on eGFR [3]. However, as AKI is defined by changes in SCr, we wanted to examine the longitudinal changes of postoperative SCr with the concept of AKI, and CKD in continuum. Moreover, eGFR can easily be calculated using SCr. Therefore, we displayed eGFR in a web application by converting the predicted SCr to predicted eGFR and then finally classifying the CKD stages of the patients. Fourth, the strong correlation between the early increase in SCr levels and CKD at 1 year after surgery, while being useful for prediction, offers little to modify treatment for improving the clinical outcome. However, our results recommend that further investigations to prevent CKD progression be focused on preventing AKI in the first place, since early SCr elevation is strongly associated with long-term clinical outcomes. Despite these limitations, our model was the first to show the serial trends of SCr during 1 year with the incorporation of preoperative, intraoperative, and postoperative information.

## Conclusions

We developed a model for predicting CKD after RN or PN, effectively extending the applicability of our prior model for predicting AKI after RN or PN [2]. The main strengths of our study were the active utilization of postoperative SCr and other laboratory values for CKD prediction and a clear demonstration of the importance of SCr measured within the first 5 days after surgery as a predictor of the 1-year SCr level. Furthermore, our web application may be helpful for patient counseling and comprehensive management planning.

## Supplementary Information

**Additional file 1: Figure S1.** The proportion of missing values among serial serum creatinine measurements preoperative (CrP), on POD 0 to 5 (Cr0, …, Cr5) and postoperative 1 month (Cr1M) (Left) and the frequency distribution of different combinations of missing values (Right) in (**A**) radical and (**B**) partial nephrectomy.

**Additional file 2: Figure S2.** Longitudinal trajectories of postoperative serum creatinine (SCr) levels in (**A**) radical and (**B**) partial nephrectomy, with the red line representing the median and the yellow band, the 95 percentiles.

**Additional file 3: Table S1.** Pairwise correlations of serum creatinine (SCr) at different time points with SCr at 1 year based on raw data prior to imputation.**Table S2.** Selected features and their associated regression coefficients of the Lasso models. Features with zero coefficients are not shown.

## Data Availability

The datasets used and/or analyzed during the current study are available from the corresponding author on reasonable request.

## Abbreviations

AKI: Acute kidney injury
BUN: Blood urea nitrogen
CBC: Complete blood count
CKD: Chronic kidney disease
eGFR: Estimated glomerular filtration rate
MAR: Missing at Random
PN: Partial nephrectomy
POD: Postoperative day
RCC: Renal cell cancer
RN: Radical nephrectomy
SCr: Serum creatinine
