# Supplementary material for: Predictive models for chronic kidney disease after radical or partial nephrectomy in renal cell cancer using early postoperative serum creatinine levels
Source: J Transl Med. 2021 Jul 16;19:307. doi: 10.1186/s12967-021-02976-2 (PMC8283951; doi:10.1186/s12967-021-02976-2)
Supplement: Supplementary file 1 — Additional file 1: Figure S1. The proportion of missing values among serial serum creatinine measurements preoperative (CrP), on POD 0 to 5 (Cr0, …, Cr5) and postoperative 1 month (Cr1M) (Left) and the frequency distribution of different combinations of missing values (Right) in (A) radical and (B) partial nephrectomy. [file 12967_2021_2976_MOESM1_ESM.docx]

**(A) Radical nephrectomy**


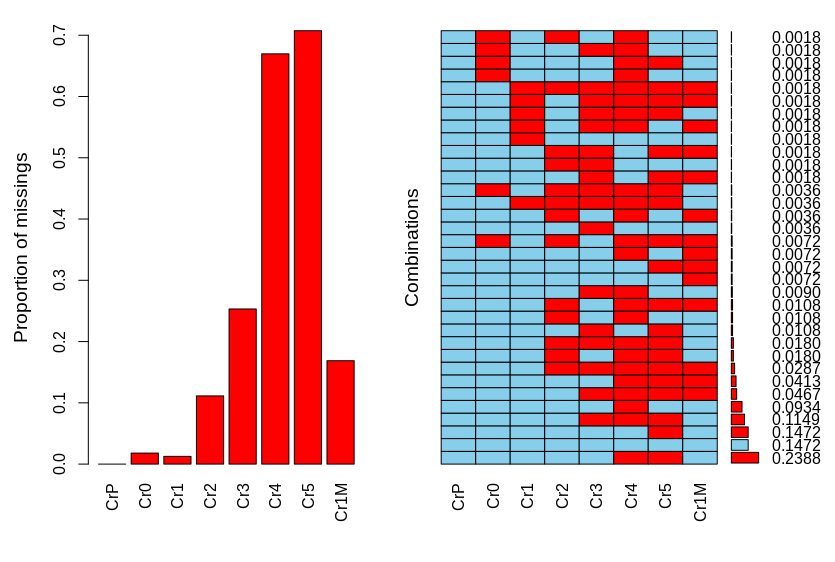


**(B) Partial nephrectomy**


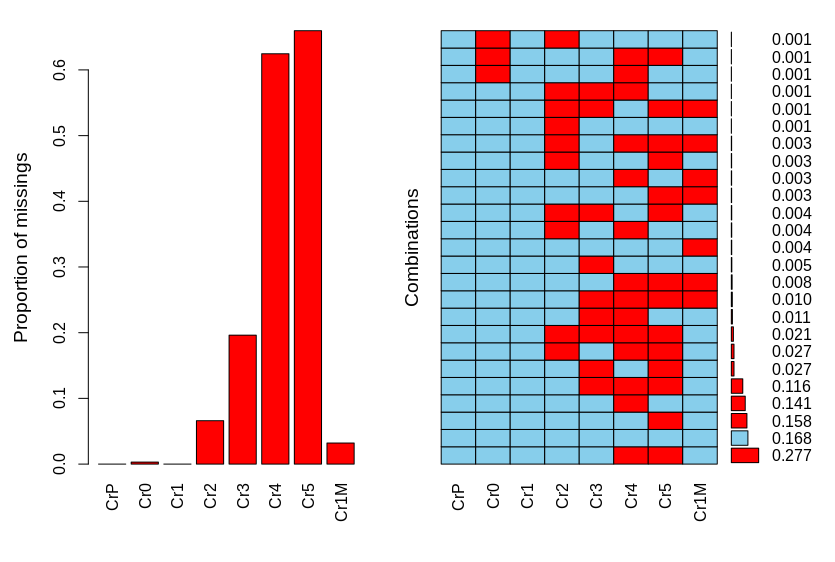


**Supplementary Figure 1.** The proportion of missing values among serial serum creatinine measurements preoperative (CrP), on POD 0 to 5 (Cr0, …, Cr5) and postoperative 1 month (Cr1M) (Left) and the frequency distribution of different combinations of missing values (Right) in (A) radical and (B) partial nephrectomy.
