# Supplementary material for: Predictive models for chronic kidney disease after radical or partial nephrectomy in renal cell cancer using early postoperative serum creatinine levels
Source: J Transl Med. 2021 Jul 16;19:307. doi: 10.1186/s12967-021-02976-2 (PMC8283951; doi:10.1186/s12967-021-02976-2)
Supplement: Supplementary file 2 — Additional file 2: Figure S2. Longitudinal trajectories of postoperative serum creatinine (SCr) levels in (A) radical and (B) partial nephrectomy, with the red line representing the median and the yellow band, the 95 percentiles. [file 12967_2021_2976_MOESM2_ESM.docx]

**(A) Radical nephrectomy**


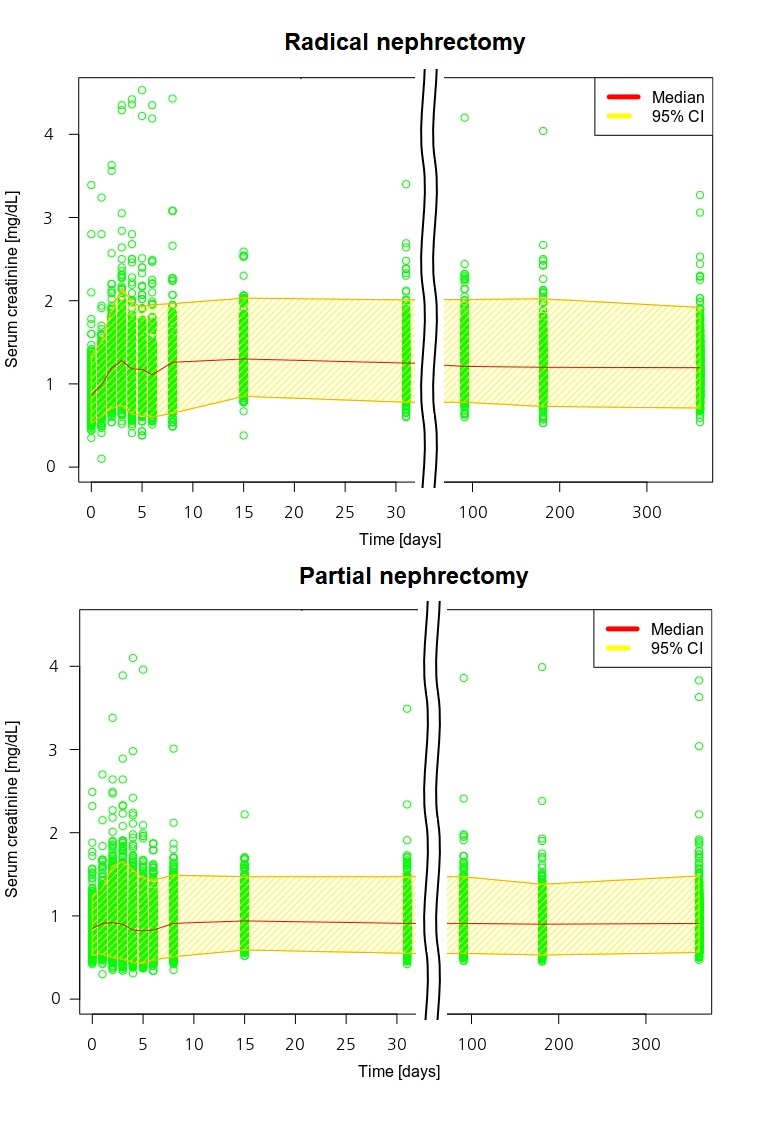


**(B) Partial nephrectomy**

**
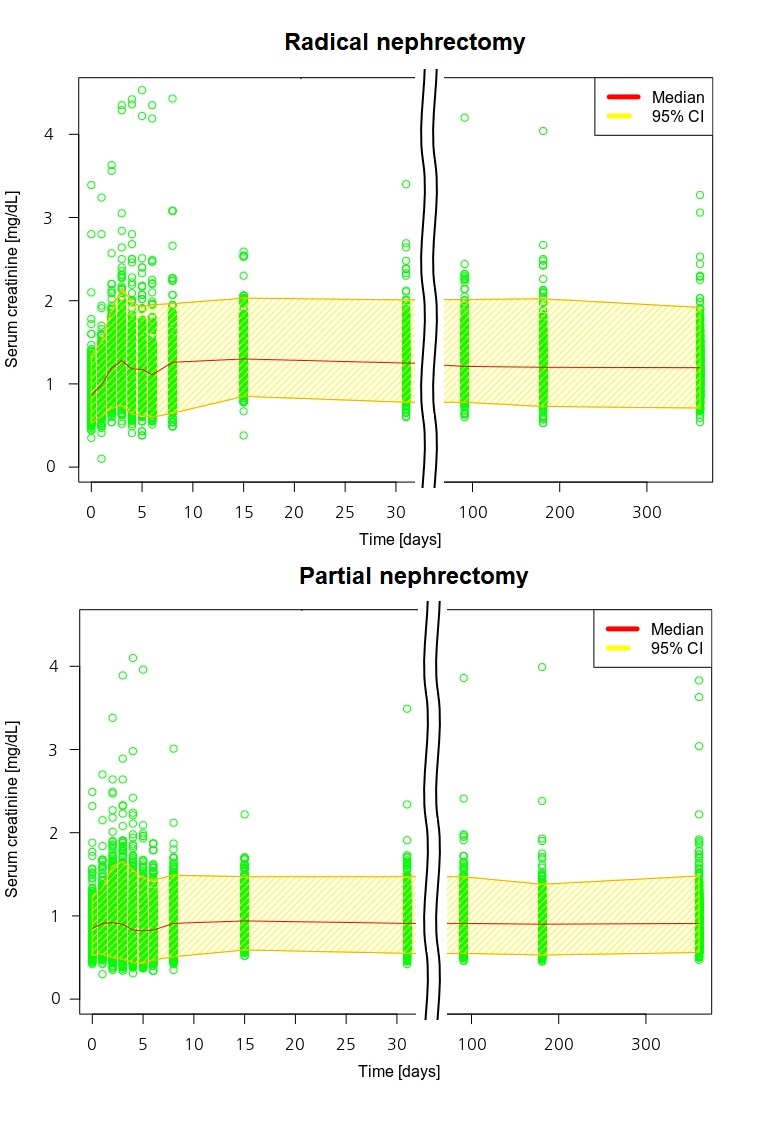
**

**Supplementary Figure 2.** Longitudinal trajectories of postoperative serum creatinine (SCr) levels in (A) radical and (B) partial nephrectomy, with the red line representing the median and the yellow band, the 95 percentiles.
