# Supplementary material for: Predictive models for chronic kidney disease after radical or partial nephrectomy in renal cell cancer using early postoperative serum creatinine levels
Source: J Transl Med. 2021 Jul 16;19:307. doi: 10.1186/s12967-021-02976-2 (PMC8283951; doi:10.1186/s12967-021-02976-2)
Supplement: Supplementary file 3 — Additional file 3: Table S1. Pairwise correlations of serum creatinine (SCr) at different time points with SCr at 1 year based on raw data prior to imputation.Table S2. Selected features and their associated regression coefficients of the Lasso models. Features with zero coefficients are not shown. [file 12967_2021_2976_MOESM3_ESM.docx]

**Supplementary Table 1.** Pairwise correlations of serum creatinine (SCr) at different time points with SCr at 1 year based on raw data prior to imputation.

|  | Preoperative | POD 0 | POD 1 | POD 2 | POD 3 | POD 4 | POD 5 | 1 month |
| --- | --- | --- | --- | --- | --- | --- | --- | --- |
| RN | r=0.8152  t=31.606 | r=0.7920  t=28.895 | r=0.7938  t=29.101 | r=0.8482  t=33.938 | r=0.8734  t=34.959 | r=0.8963  t=25.803 | r=0.9072  t=25.698 | r=0.8918  t=40.551 |
| PN | r=0.8006  t=40.262 | r=0.7968  t=39.663 | r=0.7849  t=38.17 | r=0.7955  t=38.184 | r=0.8114  t=37.476 | r=0.8412  t=28.935 | r=0.7654  t=21.043 | r=0.8521  t=48.822 |

RN, radical nephrectomy; PN, partial nephrectomy; POD, postoperative day; r, Pearson’s correlation coefficient; t, t-value (p values were <2.2e-16).

**Supplementary Table 2.** Selected features and their associated regression coefficients of the Lasso models. Features with zero coefficients are not shown.

| ${Model}_{Lasso,pre}$ | ${Model}_{Lasso,0d}$ |
| --- | --- |
| Age: 0.0648  Female gender: -0.0655  Weight: -0.0345  Hypertension: 0.0115  History of CKD: 0.558  Radical nephrectomy: 0.20  Size of mass removed: 0.182  Preoperative SCr: 0.0818  Preoperative hematocrit: -0.0367  Preoperative phosphate: -0.00398  Preoperative cholesterol: -0.0169  Preoperative albumin: -0.117  Preoperative total bilirubin: -0.0451 | Age: 0.0227  Weight: -0.0146  History of CKD: 0.0484  Radical nephrectomy: 0.0891  Size of mass removed: 0.0246  Preoperative SCr: 0.0357  Preoperative hematocrit: -0.00339  Preoperative lymphocyte: 0.00242  Preoperative potassium: 0.00136  Preoperative BUN: 0.0101  Preoperative cholesterol: -0.00891  Preoperative albumin: -0.0123  Preoperative total bilirubin: -0.00485  Preoperative systolic BP: 0.00374  Duration of anesthesia: 0.00171  $\Delta{SCr}_{0d}$: 0.069  $\Delta{hematocrit}_{0d}$: 0.00451  $\Delta{phosphate}_{0d}$: 0.00508  $\Delta{BUN}_{0d}$: 0.0219  $\Delta{total\_protein}_{0d}$: -0.000868  $\Delta{albumin}_{0d}$: -0.00178  $\Delta{ALT}_{0d}$: -0.000209 |
| ${Model}_{Lasso,1d}$ | ${Model}_{Lasso,2d}$ |
| Age: 0.0149  Female gender: -0.000925  Weight: -0.00766  History of CKD: 0.0450  Radical nephrectomy: 0.0728  Preoperative tumor size: -0.000991  Size of mass removed: 0.0114  Preoperative SCr: 0.0212  Preoperative lymphocyte: 0.0023  Preoperative phosphate: -0.000188  Preoperative cholesterol: -0.00592  Preoperative albumin: -0.0075  Preoperative alkaline phosphatase: 0.00108  Preoperative ALT: 0.00275  Preoperative total bilirubin: -0.00734  Preoperative systolic BP: 0.00255  Duration of anesthesia: 0.00373  $\Delta{SCr}_{1d}$: 0.0855  $\Delta{hematocrit}_{1d}$: 0.0152  $\Delta{monocyte}_{1d}$: -0.00643  $\Delta{potassium}_{1d}$: -0.00444  $\Delta{BUN}_{1d}$: 0.0174  $\Delta{uric\_acid}_{1d}$: 0.00539  $\Delta{ALT}_{1d}$: -0.00102  $\Delta{systolic\_BP}_{1d}$: -0.00955 | Age: 0.0130  Weight: -0.00608  History of CKD: 0.0351  Radical nephrectomy: 0.057  Size of mass removed: 0.00142  Preoperative SCr: 0.00758  Preoperative cholesterol: -0.00214  Preoperative albumin: -0.00446  Preoperative total bilirubin: -0.00482  $\Delta{SCr}_{2d}$: 0.109  $\Delta{hematocrit}_{2d}$: 0.00991  $\Delta{BUN}_{2d}$: 0.00949  $\Delta{ALT}_{2d}$: -0.00140 |
| ${Model}_{Lasso,3d}$ | ${Model}_{Lasso,4d}$ |
| Age: 0.0124  Female gender: -0.000545  Weight: -0.0117  History of CKD: 0.0337  Radical nephrectomy: 0.0515  Preoperative tumor size: -0.000034  Size of mass removed: 0.00257  Preoperative SCr: 0.00684  Preoperative monocyte: 0.00411  Preoperative potassium: 0.00315  Preoperative phosphate: -0.00491  Preoperative BUN: 0.00833  Preoperative cholesterol: -0.00319  Preoperative albumin: -0.00275  Preoperative total bilirubin: -0.00973  Preoperative systolic BP: 0.00434  Preoperative diastolic BP: 0.000731  Duration of anesthesia: 0.00255  $\Delta{SCr}_{3d}$: 0.116  $\Delta{neutrophil}_{3d}$: -0.00226  $\Delta{hematocrit}_{3d}$: -0.00678  $\Delta{BUN}_{3d}$: 0.0194  $\Delta{ALT}_{3d}$: -0.00323 | Age: 0.0119  Female gender: -0.00103  Weight: -0.0075  History of CKD: 0.0366  Radical nephrectomy: 0.0378  Size of mass removed: 0.0115  Preoperative SCr: 0.0100  Preoperative monocyte: 0.00215  Preoperative potassium: 0.00196  Preoperative phosphate: -0.00109  Preoperative BUN: 0.0178  Preoperative cholesterol: -0.00523  Preoperative albumin: -0.00285  Preoperative total bilirubin: -0.00666  Preoperative systolic BP: 0.00165  Preoperative diastolic BP: 0.00160  Duration of anesthesia: 0.00342  Amount of surgical bleeding: 0.00064  $\Delta{SCr}_{4d}$: 0.108  $\Delta{hematocrit}_{4d}$: 0.00941  $\Delta{monocyte}_{4d}$: -0.00441  $\Delta{sodium}_{4d}$: --0.00244  $\Delta{BUN}_{4d}$: 0.0327  $\Delta{uric\_acid}_{4d}$: 0.00298  $\Delta{albumin}_{4d}$: -0.00152  $\Delta{ALT}_{4d}$: -0.0031 |
| ${Model}_{Lasso,5d}$ | ${Model}_{Lasso,1m}$ |
| Age: 0.011  Weight: -0.0152  History of CKD: 0.0372  Radical nephrectomy: 0.0356  Size of mass removed: 0.00747  Preoperative SCr: 0.0208  Preoperative hematocrit: -0.000125  Preoperative monocyte: 0.00251  Preoperative BUN: 0.0220  Preoperative cholesterol: -0.00574  Preoperative albumin: -0.00986  Preoperative alkaline phosphatase: 0.00254  Preoperative total bilirubin: -0.102  Preoperative systolic BP: 0.00445  Preoperative diastolic BP: 0.000216  Duration of anesthesia: 0.00461  $\Delta{SCr}_{5d}$: 0.114  $\Delta{hematocrit}_{5d}$: 0.00748  $\Delta{calcium}_{5d}$: -0.00642  $\Delta{BUN}_{5d}$: 0.0373  $\Delta{uric\_acid}_{5d}$: 0.00525  $\Delta{ALT}_{5d}$: -0.00247  $\Delta{heart\_rate}_{5d}$: -0.000324 | Age: 0.00003  History of CKD: 0.0361  Radical nephrectomy: 0.0245  Size of mass removed: 0.00417  Preoperative SCr: 0.00858  Preoperative BUN: 0.00772  Preoperative cholesterol: -0.00089  Preoperative albumin: -0.00762  $\Delta{SCr}_{1m}$: 0.137  $\Delta{sodium}_{1m}$: -0.00123  $\Delta{BUN}_{1m}$: 0.0254  $\Delta{total\_bilirubin}_{1m}$: 0.00148 |

CKD, chronic kidney disease; SCr, serum creatinine; BUN, blood urea nitrogen; BP, blood pressure; d, day; m, month
